# Pre-Engraftment Syndrome After Autologous Stem Cell Transplantation in Relapsed or Refractory Hodgkin Lymphoma: An Association with Prior PD-1 Inhibitor Exposure

**DOI:** 10.3390/medicina62040738

**Published:** 2026-04-12

**Authors:** Dávid Tóthfalusi, Gréta Melani Csatlós, Boglárka Dobó, Fanni Borics, László Imre Pinczés, Árpád Illés, Zsófia Miltényi

**Affiliations:** 1 Division of Haematology, Department of Internal Medicine, Faculty of Medicine, University of Debrecen, 4032 Debrecen, Hungary; csatlosgretamelani@mailbox.unideb.hu (G.M.C.); dobo.boglarka@med.unideb.hu (B.D.); borics.fanni@med.unideb.hu (F.B.); pinczes.laszlo.imre@med.unideb.hu (L.I.P.); illes.arpad@med.unideb.hu (Á.I.); miltenyi.zsofia@med.unideb.hu (Z.M.); 2 Doctoral School of Clinical Medicine, University of Debrecen, 4032 Debrecen, Hungary

**Keywords:** Hodgkin lymphoma, autologous stem cell transplantation, engraftment syndrome, pre-engraftment syndrome, immune checkpoint inhibitors, PD-1 inhibitors, inflammatory complications, peri-transplant manifestations

## Abstract

*Background and Objectives*: Autologous stem cell transplantation (ASCT) remains the standard of care for relapsed or refractory Hodgkin lymphoma (R/R HL), and an increasing proportion of patients receive programmed cell death protein 1 (PD-1) inhibitors prior to transplantation. Engraftment syndrome (ES) is a noninfectious inflammatory complication classically associated with neutrophil recovery; however, early peri-transplant inflammatory manifestations remain poorly characterized and may mimic infectious complications. We aimed to evaluate peri-transplant inflammatory events after ASCT, with particular emphasis on ES-compatible manifestations occurring before neutrophil engraftment and their association with prior PD-1 inhibitor exposure. *Materials and Methods*: In this single-center retrospective cohort study, 64 consecutive adult patients with HL undergoing ASCT between 2018 and 2025 were analyzed. ES was defined according to Spitzer and Maiolino criteria. Inflammatory manifestations fulfilling these criteria but occurring prior to neutrophil recovery were classified as pre-engraftment syndrome (pre-ES). Clinically significant events were defined by the requirement for systemic corticosteroid therapy. Clinical and laboratory parameters were compared using non-parametric statistical analyses. *Results*: No cases fulfilled the Spitzer criteria for classical ES, while three patients (4.7%) met the Maiolino criteria, none requiring corticosteroid therapy. Using the broader Maiolino definition, pre-ES was observed in 34 patients (53.1%) when the conventional engraftment time window was disregarded; however, only three patients required systemic corticosteroid therapy. Importantly, all three cases also fulfilled the Spitzer criteria outside the conventional time window, whereas the remaining Maiolino-defined pre-ES cases were self-limiting. All steroid-requiring pre-ES cases occurred exclusively in PD-1-exposed patients, and prior PD-1 therapy was significantly associated with severe pre-ES (*p* = 0.0007), although this finding is based on a very small number of events. These patients also demonstrated significantly higher early C-reactive protein (CRP) levels. *Conclusions*: While classical ES after ASCT was uncommon, clinically significant pre-ES occurred exclusively in PD-1-exposed patients. These early inflammatory events may represent a distinct phenotype and require prompt recognition and timely corticosteroid therapy after exclusion of infection. Prospective studies are warranted to validate these findings and refine risk stratification and monitoring strategies.

## 1. Introduction

Classical Hodgkin lymphoma (HL) is highly curable, with approximately 80–90% of young patients achieving long-term remission after first-line therapy; nevertheless, 10–30% of patients experience relapse or refractory disease (R/R). Salvage chemotherapy followed by autologous stem cell transplantation (ASCT) remains the standard of care in R/R patients. After ASCT, reported 5-year overall survival (OS) rates reach up to 86%, whereas 5-year progression-free survival (PFS) ranges from approximately 56% to 66% [1,2].

Factors influencing the success of ASCT include, in part, disease characteristics at relapse (primary chemorefractory disease, stage IV at relapse, extranodal involvement, B symptoms); however, the most important factor is achieving complete metabolic remission (CMR) before ASCT [3]. In this regard, newly available innovative therapies that can be administered even before transplantation—such as brentuximab vedotin (BV) and programmed cell death protein 1 (PD-1) inhibitors—play a crucial role in increasing the likelihood of achieving CMR.

Engraftment syndrome (ES) is a noninfectious, systemic inflammatory complication that occurs during the peri-engraftment period following hematopoietic stem cell transplantation (HSCT), most commonly after autologous transplantation [4].

ES frequently manifests with clinical features that overlap with those of early peri-transplant complications, including infectious fever, drug-related toxicity, fluid overload, and—particularly in the allogeneic setting—early graft-versus-host disease (GVHD). As a result, its differential diagnosis is often challenging. The identification of ES is primarily based on its close temporal relationship with neutrophil recovery, the lack of an identifiable infectious etiology, and a rapid clinical improvement following the initiation of corticosteroid therapy. To facilitate a more uniform diagnostic approach, several diagnostic criteria have been proposed, with the Spitzer and Maiolino criteria being the most commonly used [4,5].

The Spitzer criteria, introduced in 2001, define ES as the presence of either three major manifestations—noninfectious fever, a maculopapular rash involving more than 25% of the body surface area, and noncardiogenic pulmonary edema or hypoxemia—or two major manifestations accompanied by at least one minor criterion. Minor features include weight gain exceeding 2.5% of baseline body weight, hepatic or renal dysfunction, and transient encephalopathy. All clinical signs must develop within 96 h of neutrophil engraftment [6].

In 2003, Maiolino and colleagues proposed a simplified diagnostic definition, requiring noninfectious fever in combination with at least one additional clinical feature, such as skin rash, pulmonary infiltrates, or diarrhea. According to this definition, symptoms should occur within a narrower time frame, ranging from 24 h before to 48 h after the appearance of neutrophils in the peripheral blood [7]. The main diagnostic features of both systems are summarized in Table 1.

With the increasing use of immune checkpoint inhibitors (ICI), an increasing number of patients with R/R HL are exposed to PD-1 inhibitors prior to ASCT. Although this strategy has substantially improved disease control and transplant outcomes, concerns have emerged regarding immune-mediated toxicities in the peri-transplant period, including a potentially increased risk of ES. Because data on the incidence, clinical spectrum, and severity of ES—particularly ES-compatible inflammatory manifestations arising before neutrophil recovery—remain limited and heterogeneous, we conducted a retrospective cohort analysis to evaluate ES-related inflammatory complications after ASCT, with special emphasis on the impact of prior PD-1 inhibitor exposure and on identifying factors associated with clinically significant, steroid-requiring disease.

Pre-engraftment inflammatory manifestations temporally preceding neutrophil recovery are insufficiently defined in the current ES literature and are not fully captured by conventional Spitzer or Maiolino diagnostic criteria. In the present study, we analyzed such events separately and use the term pre-engraftment syndrome (pre-ES) as a descriptive label for ES-compatible inflammatory manifestations occurring before neutrophil engraftment. We do not intend to propose pre-ES as a validated diagnostic entity; rather, we use the term to describe an observed clinical pattern that may be relevant in PD-1-exposed patients and that warrants further prospective study.

Several alternative explanations for these manifestations must be carefully considered, particularly in the early peri-transplant setting where clinical features are often nonspecific and overlapping. Although extensive microbiological and clinical evaluation did not identify a causative pathogen in clinically significant cases, occult or culture-negative infection cannot be definitively excluded, especially in neutropenic patients and in the context of prior or ongoing antimicrobial therapy. In addition, the observed symptoms may reflect noninfectious inflammatory processes unrelated to classical ES, including drug-related toxicity (e.g., conditioning regimen-associated mucosal or pulmonary injury), granulocyte colony-stimulating factor (G-CSF)-associated cytokine-mediated inflammatory reactions, capillary leak phenomena related to endothelial dysfunction, or immune-mediated toxicity in the context of prior PD-1 inhibitor exposure. Furthermore, early peri-transplant complications such as incipient peri-engraftment respiratory distress syndrome (PERDS) or other forms of immune reconstitution-associated inflammation may present with partially overlapping clinical features. However, PERDS is typically characterized by predominant and often severe pulmonary involvement with hypoxemia, whereas the inflammatory manifestations observed in our cohort were more heterogeneous and not uniformly dominated by respiratory failure. In addition, cytokine release syndrome (CRS), although sharing features of systemic hyperinflammation, is most commonly associated with cellular therapies and is characterized by pronounced cytokine-driven hemodynamic instability, which was not a defining feature in our patients. Given the retrospective design of the study and the absence of systematic biomarker or cytokine profiling, precise differentiation between these entities remains inherently limited.

Within these limitations, our observations suggest that pre-ES manifestations may occur more frequently in PD-1-exposed patients and, in a subset of cases, may precede clinically significant, steroid-requiring inflammatory manifestations. This potential association underscores the importance of heightened clinical awareness, careful differential diagnosis, and timely initiation of corticosteroid therapy once infectious causes have been reasonably excluded.

Accordingly, the concept of pre-ES should be interpreted as a descriptive observation that generates a clinical and biological hypothesis regarding early peri-transplant inflammatory manifestations, rather than as evidence for a distinct diagnostic category.

## 2. Materials and Methods

A retrospective cohort analysis was conducted including adult patients (≥18 years) with HL who underwent ASCT at our department between 1 January 2018 and 31 December 2025. The study was conducted in accordance with the Declaration of Helsinki and was approved by the Institutional Review Board of the University of Debrecen (DE RKEB/IKEB 6477-2023, 21 June 2023).

The primary objective of the study was to evaluate the occurrence of ES-related inflammatory manifestations in this patient population and to explore clinical and transplant-related factors associated with the development and severity of these complications, with particular focus on cases requiring systemic corticosteroid therapy.

All patients received uniform conditioning with carmustine, etoposide, cytarabine, and melphalan (BEAM), followed by autologous stem cell infusion. G-CSF was administered routinely from day +1 until neutrophil engraftment. In patients presenting with inflammatory symptoms during the peri-transplant period, a standardized diagnostic workup was performed to exclude infectious etiologies. This evaluation included repeated sets of peripheral and central venous blood cultures, urine cultures, and targeted microbiological sampling based on clinical presentation. Imaging studies, including chest X-ray or computed tomography (CT), were performed in patients with respiratory symptoms or persistent fever. In addition, viral diagnostics using polymerase chain reaction (PCR)-based assays (e.g., for respiratory viruses, cytomegalovirus (CMV), and Epstein–Barr virus (EBV)), as well as serum beta-D-glucan testing, were performed according to institutional protocols. Empirical broad-spectrum antimicrobial therapy was initiated promptly in all patients with neutropenic fever, in accordance with international guidelines, and subsequently adjusted based on clinical evolution, microbiological findings, and suspected source of infection. Initial empirical regimens typically consisted of an anti-pseudomonal beta-lactam agent (such as ceftazidime or meropenem), frequently in combination with an aminoglycoside (e.g., amikacin), with the addition of vancomycin in selected cases based on clinical suspicion of Gram-positive infection or catheter-related complications. Antifungal or antiviral therapy was introduced when clinically indicated. Inflammatory manifestations were considered noninfectious only after comprehensive evaluation failed to identify a causative pathogen and in the absence of clinical response to appropriate antimicrobial therapy. Systemic corticosteroids were initiated only after reasonable exclusion of infectious etiologies and persistent or progressive symptoms despite 48–72 h of antimicrobial treatment.

ES was defined according to the diagnostic criteria proposed by Spitzer and Maiolino. Inflammatory manifestations fulfilling these criteria but occurring before neutrophil engraftment were analyzed separately and descriptively classified as pre-ES. It should be emphasized that this classification does not represent a validated extension of the original Spitzer or Maiolino definitions; rather, these criteria were applied outside their predefined temporal window solely for descriptive purposes, in order to capture early inflammatory manifestations.

Severe disease was defined a priori as inflammatory symptoms requiring systemic corticosteroid therapy. Clinical data were extracted from electronic medical records and institutional transplant databases. Variables analyzed included demographic characteristics, disease status before ASCT, prior exposure to PD-1 inhibitors, the time interval between the last PD-1 dose and transplantation, reinfused CD34+ cell dose and mononuclear cell (MNC) count, time to symptom onset, time to neutrophil engraftment, the spectrum of ES-compatible clinical manifestations, and laboratory markers of systemic inflammation, including serial C-reactive protein (CRP) measurements. Patients meeting Maiolino-based criteria for pre-ES, as well as those fulfilling Spitzer criteria independent of the engraftment time window, were further stratified into steroid-requiring (severe) and non-steroid-requiring (non-severe) subgroups for comparative analyses.

Given the limited sample size and the non-normal distribution of most variables, statistical analyses were performed using non-parametric methods. Continuous data are presented as medians with interquartile ranges and were compared using the Mann–Whitney U test, while categorical variables were analyzed using Fisher’s exact test. All tests were two-sided, and a *p* value < 0.05 was considered statistically significant. Owing to the exploratory nature of the study and the relatively small cohort, no formal correction for multiple testing was applied.

No formal exclusion criteria were applied; all consecutive eligible patients meeting the inclusion criteria were included in the analysis to minimize selection bias and reflect real-world clinical practice.

## 3. Results

### 3.1. Patient Characteristics and Treatment

During the study period, 64 patients with HL (31 females and 33 males) underwent ASCT. The median age at the time of transplantation was 32 years (range, 18–62). Among the 64 patients included in the cohort, 24 had primary refractory disease, whereas 40 underwent ASCT for relapsed HL. The time interval from initial diagnosis to ASCT ranged from 5 to 185 months, with a mean of 26 months and a median of 15 months, reflecting the heterogeneity of disease course prior to transplantation. The estimated 5-year OS and PFS rates were 90% and 87%, respectively.

First-line therapy consisted of doxorubicin, bleomycin, vinblastine, and dacarbazine (ABVD) in 63 patients, while one patient received brentuximab vedotin (BV) in combination with doxorubicin, vinblastine, and dacarbazine (AVD). All patients underwent salvage therapy prior to ASCT, initially with two cycles of dexamethasone, high-dose cytarabine, and cisplatin (DHAP), which enabled successful peripheral blood stem cell mobilization and collection.

In patients who failed to achieve CMR on post-salvage positron emission tomography/computed tomography (PET/CT), subsequent treatment consisted of BV in combination with bendamustine or ifosfamide, carboplatin, and etoposide (ICE) (34 patients), followed by pembrolizumab in cases with persistent metabolic activity (8 patients). Among PD-1-exposed patients, six received pembrolizumab and two received nivolumab. The number of treatment cycles was a median of 4.5 (range 2 to 7). Based on standard dosing intervals (every 3 weeks for pembrolizumab and every 2 weeks for nivolumab), the estimated duration of PD-1 inhibitor therapy was a median of 12 weeks (range 6 to 21). No prior immune-related adverse events (irAEs) were documented in this subgroup. These latter patients subsequently proceeded to ASCT after PD-1 inhibitor therapy, with a median interval of 41 days (range, 30–146) between the last dose and transplantation. At the time of ASCT, 54 patients were in CMR, nine were in partial remission, and one patient had stable disease. The detailed baseline, disease, and treatment characteristics of the cohort are summarized in Table 2.

### 3.2. Engraftment Syndrome by Standard Criteria

According to the Spitzer criteria, no cases of ES were observed. Using the Maiolino criteria, ES was diagnosed in 3 patients (4.7%), none of whom required systemic corticosteroid therapy.

### 3.3. Pre-Engraftment Syndrome Manifestations

**Figure 1:**
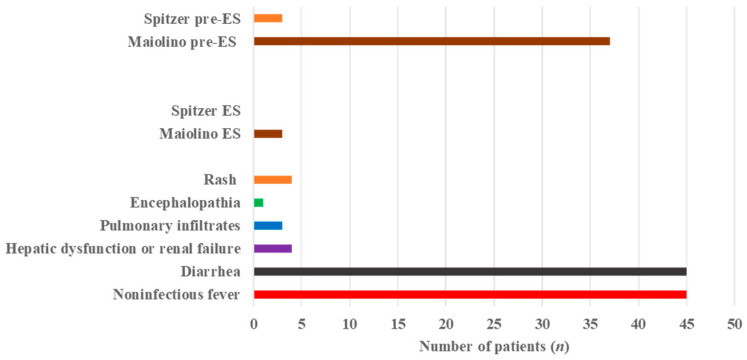
Frequency of engraftment syndrome-related clinical manifestations and diagnostic classifications in the study cohort. ES: engraftment syndrome; pre-ES: pre-engraftment syndrome.

When the time-to-engraftment criterion was disregarded, pre-ES manifestations fulfilling the Maiolino criteria outside their original temporal framework were identified in 34 patients (53.1%). Among these, three patients required systemic corticosteroid therapy and were therefore classified as having severe pre-ES. These same three cases also fulfilled the Spitzer criteria outside the conventional engraftment time window. In contrast, the remaining Maiolino-defined pre-ES cases were self-limiting and did not require corticosteroid treatment. All steroid-requiring pre-ES cases had received PD-1 inhibitor therapy prior to ASCT. The distribution of ES-compatible inflammatory manifestations in the overall cohort is summarized in Figure 1.

### 3.4. Clinical Characteristics of Pre-Engraftment Syndrome

The median time to symptom onset in the pre-ES cohort was 4 days (range, 2–9). Within the pre-ES group, symptom onset occurred at a median of 4 days in steroid-requiring (severe pre-ES) cases and 5 days (range, 2–9) in non-steroid-requiring (non-severe pre-ES) cases. Symptom onset was not assessed in patients without pre-ES, as this parameter is inherently linked to the presence of pre-ES-related clinical manifestations. In the overall cohort, the median time to neutrophil engraftment was 9 days (range, 6–17). Patients without pre-ES showed a comparable engraftment time (median 9 days, range, 6–17), whereas slightly longer engraftment times were observed in severe pre-ES cases (median 10 days, range, 8–17) compared with non-severe pre-ES cases (median 9 days, range, 6–14).

### 3.5. Stem Cell Graft Characteristics

The median number of reinfused CD34+ cells in the overall cohort was 5.14 × 10^6^/kg (range 2.54–8.44). Patients who developed severe, steroid-requiring pre-ES received a numerically higher median CD34+ cell dose compared with patients with non-severe, self-limiting pre-ES (7.88 × 10^6^/kg vs. 5.08 × 10^6^/kg, respectively). Patients without pre-ES had a comparable median CD34+ cell dose of 5.36 × 10^6^/kg (range, 2.54–8.56).

The median reinfused MNC dose was 3.29 × 10^8^/kg (range, 0.12–16.6). In contrast to CD34+ cell counts, patients with severe pre-ES received lower median MNC doses than those with non-severe pre-ES (1.67 × 10^8^/kg vs. 3.3 × 10^8^/kg). However, none of the observed differences in stem cell or MNC doses between the severe and non-severe pre-ES groups reached statistical significance when analyzed using the Mann–Whitney U test. A detailed comparison of baseline, laboratory, and transplant-related characteristics between severe and non-severe pre-ES cases is presented in Table 3.

### 3.6. Association Between Pre-ES and Prior PD-1 Inhibitor Exposure

Fisher’s exact test demonstrated a statistically significant association between prior PD-1 inhibitor exposure and Spitzer-defined pre-ES (*p* = 0.0007). PD-1-exposed patients showed a higher risk of developing steroid-requiring pre-ES; however, given the small number of events, these findings should be interpreted with caution, as the limited number of clinically significant cases restricts statistical power and may lead to overestimation of associations.

No significant differences were observed between the steroid-requiring (severe) and non-severe pre-ES groups across the evaluated baseline, laboratory, and transplant-related variables. Demographic and disease characteristics, including age, sex, and disease status at ASCT, were comparable between groups. Markers of hematologic recovery (peripheral blood counts) did not differ significantly. Transplant-related parameters, including time to neutrophil engraftment, reinfused CD34+ cell dose, MNC dose, and timing of symptom onset, also showed no statistically significant associations with disease severity. Owing to the limited sample size and the small number of steroid-requiring cases, these comparisons should be considered exploratory and interpreted cautiously.

### 3.7. Inflammatory Marker Dynamics (C-Reactive Protein)

**Figure 2:**
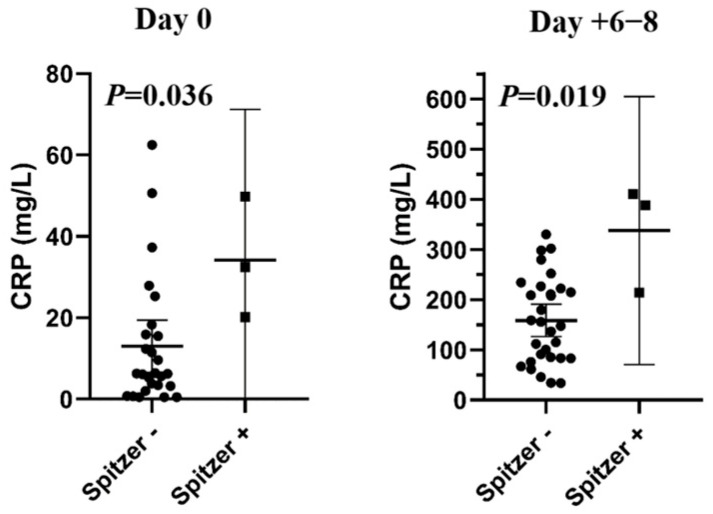
C-reactive protein (CRP) levels in patients with and without Spitzer-defined pre-engraftment syndrome at day 0 (the day of stem cell infusion) and days +6–8 (around the time of neutrophil engraftment) after autologous stem cell transplantation (ASCT).

Longitudinal analysis of CRP levels demonstrated significantly higher CRP values in patients fulfilling Spitzer-defined pre-ES criteria already at day 0 (the day of stem cell infusion) compared with patients without pre-ES (*p* = 0.036). This difference persisted during days 6–8 post-transplantation, around the time of neutrophil engraftment, with CRP levels remaining significantly elevated in the Spitzer-defined pre-ES group (*p* = 0.019) (Figure 2). These findings suggest an early inflammatory response in clinically significant pre-ES rather than delayed infectious complications; however, as CRP is a nonspecific marker, they do not provide direct insight into the underlying immunological mechanisms or cytokine dynamics.

Overall, while pre-ES was frequent when broader diagnostic criteria were applied, clinically significant, steroid-requiring pre-ES was rare and occurred exclusively in patients previously exposed to PD-1 inhibitor therapy. No other baseline or transplant-related characteristics showed significant differences between groups, although these comparisons were limited by the small number of events.

## 4. Discussion

### 4.1. Principal Findings

In this single-center retrospective cohort study, we demonstrate that clinically significant ES-compatible inflammatory manifestations may occur prior to neutrophil recovery, representing a distinct pre-engraftment inflammatory phenotype. Importantly, all steroid-requiring cases were observed exclusively in patients previously exposed to PD-1 inhibitors, suggesting a possible association between checkpoint blockade and early peri-transplant immune dysregulation.

No cases of classical ES were identified according to the Spitzer criteria within the conventional peri-engraftment window, while three patients fulfilled Maiolino criteria, all with clinically mild and self-limiting disease. In contrast, when the temporal criterion linked to neutrophil engraftment was disregarded, three patients met Spitzer diagnostic criteria prior to neutrophil recovery and were therefore classified as pre-ES; importantly, all of these cases required systemic corticosteroid treatment. Using the broader Maiolino definition, pre-ES manifestations were observed in over half of the cohort; however, only three cases were clinically significant and steroid-requiring, all of which met Spitzer criteria and were classified as pre-ES, whereas the remaining Maiolino-defined cases were self-limiting.

These findings highlight an important limitation of current ES definitions, which are inherently linked to neutrophil recovery and may therefore fail to capture early peri-transplant inflammatory manifestations. The concept of pre-ES, as defined in our study, provides a clinically meaningful framework for recognizing inflammatory events occurring before engraftment that share key features with classical ES but differ in their timing. Taken together, our results indicate that the clinically most relevant inflammatory events in this cohort did not occur at the time of neutrophil recovery, as expected for classical ES, but rather in the pre-engraftment phase.

Notably, all steroid-requiring pre-ES cases occurred exclusively in patients previously exposed to PD-1 inhibitors, supporting a specific association between PD-1 inhibitor exposure and clinically relevant peri-transplant inflammatory manifestations. No other baseline or transplant-related characteristics differentiated severe from non-severe cases. Moreover, patients with clinically significant pre-ES demonstrated early and sustained CRP elevation, consistent with a distinct inflammatory phenotype rather than delayed infectious complications.

Our findings should be interpreted in light of both the strengths and limitations of the study. Strengths include the relatively homogeneous disease population, the uniform use of BEAM conditioning, consistent single-center supportive care practices, and the systematic assessment of peri-transplant inflammatory events using both Spitzer and Maiolino diagnostic frameworks. In addition, by explicitly analyzing ES-compatible manifestations outside the conventional engraftment window, we were able to identify and characterize an underrecognized pre-engraftment inflammatory pattern with potential clinical relevance. At the same time, the retrospective single-center design, the limited cohort size, and the very small number of steroid-requiring events restrict statistical power and preclude robust multivariable modeling. Furthermore, cytokine profiling was not routinely available, and the term pre-ES was used descriptively rather than as a validated diagnostic entity. These considerations underscore the exploratory nature of the present analysis and the need for prospective validation.

### 4.2. Role of ASCT Following Contemporary Salvage Treatments in HL

Although previous studies have questioned whether ASCT is always necessary in the treatment of R/R patients, particularly with the integration of ICI into salvage therapy, early data suggest that favorable outcomes cannot be achieved without ASCT in all cases. Moskowitz et al. treated 24 patients without ASCT using pembrolizumab in combination with gemcitabine, vinorelbine, and pegylated liposomal doxorubicin (GVD), followed by pembrolizumab maintenance. After a median follow-up of 23.4 months, the 2-year PFS was 51%. Patients who subsequently relapsed or were refractory underwent ASCT as third-line therapy, and stage IV disease was associated with a higher likelihood of requiring transplantation [8].

Treatment with BV as monotherapy without subsequent ASCT does not result in durable survival. In R/R patients, outcomes with BV alone are generally modest in terms of durability. In a Turkish 14-center retrospective study by Bakirtas et al. including 82 patients with R/R HL treated with BV monotherapy, those who proceeded to ASCT after BV had a significantly longer median OS compared with patients who did not undergo transplantation (19.6 vs. 7.8 months; *p* < 0.001) [9].

Previous studies, including multicenter retrospective analyses of heavily pretreated HL patients who received PD-1 blockade prior to ASCT, have demonstrated excellent outcomes, with high PFS and OS rates. In the study by Merryman et al., PD-1 inhibitor therapy was administered before ASCT in 78 patients, resulting in an 18-month PFS of 81% and an OS of 96%. Favorable outcomes were observed even among patients with chemorefractory disease or positive pre-ASCT PET/CT findings. This observation may be explained by the chemosensitizing effect of PD-1 blockade, false-positive PET/CT results, or pseudoprogression, which has been described in other studies. In contrast, patients who did not respond to PD-1 inhibitor therapy had inferior outcomes, with an 18-month PFS of only 51% [10].

### 4.3. Clinical Presentation and Pathophysiology of ES

The onset of ES typically parallels neutrophil recovery and reflects an exaggerated inflammatory response associated with increased capillary permeability. Clinically, ES is characterized by noninfectious fever, erythematous or maculopapular skin rash, diarrhea, and signs of capillary leak, including rapid weight gain, generalized edema, and hypoalbuminemia. Pulmonary involvement, presenting as noncardiogenic pulmonary infiltrates and hypoxemia, represents a severe manifestation of the syndrome and may progress to PERDS [4].

The pathophysiology of ES remains incompletely understood. Current evidence suggests that during neutrophil recovery following HSCT, an exaggerated inflammatory response occurs, driven by the release of proinflammatory cytokines, including interleukins (IL-1, IL-2, IL-6), tumor necrosis factor-alpha (TNF-α), and interferon-gamma (IFN-γ), as well as the generation of reactive oxygen species, neutrophil degranulation products, and endothelial activation. This cytokine-mediated cascade results in increased capillary permeability, tissue edema, and a systemic inflammatory response. In addition, ES appears to share pathogenic features with GVHD-like immune reactions, likely related to transient immune dysregulation and aberrant T-cell activation during early immune reconstitution, even in the absence of alloantigen mismatch [5,11].

Importantly, the pathophysiology and clinical context of ES differ between autologous and allogeneic transplantation settings. In the autologous setting, ES is generally considered a cytokine-driven inflammatory process associated with neutrophil recovery, endothelial activation, and transient immune dysregulation. In contrast, in the allogeneic setting, early inflammatory manifestations may overlap with or represent early forms of acute GVHD, reflecting donor-recipient immune interactions and alloimmune activation.

Clinically, this distinction has important implications. While ES after autologous transplantation is typically self-limiting or responsive to corticosteroids, inflammatory complications in the allogeneic setting may follow a more complex course, with a higher risk of progression to clinically significant GVHD. These differences highlight that, although ES-like manifestations may share certain clinical features across transplant settings, their underlying mechanisms and clinical consequences are not fully equivalent [4,5].

However, the classical definition of ES is intrinsically linked to neutrophil engraftment, which may limit its applicability in cases where inflammatory manifestations arise earlier in the transplant course. In our cohort, clinically significant inflammatory events were observed prior to neutrophil recovery and frequently required systemic corticosteroid therapy.

Rather than definitively representing a distinct pathophysiological entity, these findings highlight that clinically relevant inflammatory complications may occur outside the conventional temporal window of ES. This has important implications for clinical recognition, as such early manifestations may not be readily identified within existing diagnostic frameworks.

In this context, the concept of a pre-ES may serve as a useful clinical construct to describe inflammatory events occurring before neutrophil recovery. Although this entity remains insufficiently defined in the literature, its recognition is of practical importance, as early inflammatory symptoms in the pre-engraftment phase may otherwise be misattributed—most notably to infectious complications.

Importantly, our findings emphasize that the key issue may not necessarily be a fundamentally different mechanism, but rather the timing and clinical recognition of these events. Early identification of pre-ES may facilitate timely initiation of immunomodulatory therapy, potentially improving patient outcomes and preventing progression to more severe complications.

### 4.4. Incidence of ES in Heterogeneous Transplant Cohorts

Against this heterogeneous and evolving background, we conducted a detailed single-center analysis of ES and ES-compatible inflammatory manifestations in a contemporary cohort of patients with R/R HL undergoing ASCT. In contemporary clinical practice in R/R HL, the increasing use of PD-1 inhibitors prior to transplantation has been accompanied by the emergence of peri-transplant inflammatory manifestations that are not fully captured by conventional ES definitions. In particular, inflammatory manifestations occurring before neutrophil engraftment—commonly underrecognized and inconsistently reported in the literature—may represent a distinct and clinically relevant entity. By applying both Spitzer and Maiolino diagnostic criteria and extending the analysis to inflammatory events occurring before neutrophil engraftment, we systematically evaluated the occurrence, timing, and clinical relevance of ES-related inflammatory manifestations across the peri-transplant period. This approach allowed a nuanced characterization of peri-transplant inflammatory complications, with particular emphasis on clinically significant, steroid-requiring pre-ES and its association with prior PD-1 inhibitor exposure, and enabled contextualization of our findings within the existing literature.

Before focusing specifically on HL-directed studies, our findings should be interpreted within the broader landscape of ES across heterogeneous HSCT populations. Published analyses of mixed autologous and allogeneic cohorts have reported markedly variable ES incidences, typically ranging from approximately 5% to over 30%, depending on the diagnostic framework applied (Table 4). Reported rates are strongly influenced by the use of stringent criteria, such as Spitzer, versus broader definitions, such as Maiolino, as well as by differences in underlying diseases, conditioning regimens, graft characteristics, and supportive care practices. Consequently, direct comparison across studies is limited by substantial methodological heterogeneity.

Importantly, most of these cohorts were assembled prior to the widespread integration of PD-1 inhibitors into salvage strategies for HL. They therefore reflect the clinical spectrum of ES in an immunologically distinct therapeutic era and provide a necessary reference framework. However, they do not fully capture the evolving peri-engraftment inflammatory phenotypes that may emerge in PD-1-exposed populations.

### 4.5. Peri-Engraftment Inflammatory Complications in Heterogeneous Cohorts Including HL

Data on the incidence and clinical characteristics of ES in patients with HL undergoing ASCT remain limited and heterogeneous. Earlier reports by Maiolino, Bai, and Sheth [7,15,16] described ES incidence rates ranging from approximately 5–10% to as high as 33%; however, direct comparison across studies is challenging due to substantial differences in diagnostic criteria, patient populations, and study endpoints. In addition, Bai et al. focused primarily on PERDS rather than classical ES, and their cohort already included patients treated with PD-1 inhibitors. Interpretation of the study by Sheth et al. is further complicated by inconsistencies in ES classification, as a considerable proportion of patients did not fulfill either the Maiolino or Spitzer criteria, and incomplete clinical data were reported for at least one case.

Our findings are consistent with the large single-center analysis by Carreras et al. [17], who reported ES in 42 of 328 patients (12.8%) undergoing ASCT, including 48 patients with HL, among whom only two cases of ES were observed (4.1%). In that study, patients with ES exhibited a pronounced inflammatory response, reflected by a markedly higher mean CRP level compared with patients without ES (17.5 ± 7.3 vs. 2.4 ± 3.4; *p* = 0.0001). Similarly, we detected significantly elevated CRP levels already at day 0 and during the early post-transplant period in patients with clinically significant, steroid-requiring pre-ES. CRP concentrations were significantly higher in Spitzer-defined pre-ES cases both at day 0 (*p* = 0.036) and at days +6–8 (*p* = 0.019), indicating an early-onset and sustained inflammatory response preceding neutrophil engraftment. Together, these data support the concept that classical ES after ASCT is relatively uncommon in HL, whereas inflammatory complications in the peri-engraftment period—particularly those occurring before neutrophil recovery—remain clinically relevant and are characterized by an early and sustained inflammatory response rather than delayed infectious complications.

More recent studies have specifically addressed the impact of PD-1 inhibitor exposure on peri-transplant inflammatory manifestations, particularly PERDS, a severe inflammatory complication distinct from but related to classical ES. Bai et al. [15] reported a PERDS incidence of 6.1% in a cohort of 260 patients undergoing ASCT, including 37 patients with HL, among whom four cases of PERDS were observed. Notably, prior anti-PD-1 therapy emerged as a significant risk factor for peri-transplant inflammatory complications: patients previously exposed to PD-1 inhibitors showed a markedly higher incidence of ES-related inflammatory events compared with PD-1-naïve patients (48.3% vs. 16.7%). Inflammatory marker analysis in that study further supported enhanced immune activation in affected patients. CRP levels were significantly higher in the PERDS group on post-transplant days 1, 5, and 10, and a greater proportion of patients exhibited elevated IL-6 and IL-10 concentrations on day 10. Among PD-1-exposed patients, the incidence of PERDS was significantly increased, IL-10 elevation at day 10 was more frequent, and transplant-related mortality was significantly higher compared with patients who had not received PD-1 inhibitor therapy. Collectively, these findings highlight a state of sustained cytokine dysregulation and heightened inflammatory responsiveness in PD-1-exposed patients during the peri-engraftment period. Key studies reporting ES incidence and clinical characteristics in mixed-diagnosis HSCT cohorts that included HL subpopulations are summarized in Table 5.

### 4.6. Impact of Prior PD-1 Inhibitor Exposure on Peri-Engraftment Inflammation

Taranto et al. [18] reported ES in approximately 20% of patients previously treated with PD-1 inhibitors, a rate substantially higher than observed in our cohort. In their analysis, several potential risk factors—including the number of infused stem cells, duration of ICI exposure, timing of ICI therapy in relation to autologous transplantation, and plerixafor use—were systematically evaluated but did not reach statistical significance. These findings suggest that conventional transplant-related parameters alone may not adequately explain the increased susceptibility to ES observed in PD-1-exposed patients.

In contrast, Khouderchah et al. [19] reported a lower overall ES incidence of 2.8%; however, their study highlighted a broader spectrum of irAEs occurring within the first 30 days after ASCT in patients previously exposed to checkpoint inhibitors. These events included not only ES but also auto-GVHD-like phenomena and other immune-mediated toxicities such as pneumonitis, colitis, and hepatitis. Patients with irAEs prior to ASCT appeared particularly vulnerable, as several experienced peri-transplant immune complications, including flares of prior toxicities or ES. This observation underscores a heightened susceptibility to peri-transplant immune dysregulation in this subgroup and raises the possibility that tailored prophylactic strategies—such as cautious use or avoidance of post-transplant G-CSF—may be warranted in selected high-risk patients.

Further supporting the association between PD-1 inhibitor exposure and ES, Odetola et al. [20] reported an overall ES incidence of 36% based on the Maiolino criteria, with a markedly higher rate among PD-1 inhibitor-exposed patients compared with PD-1-naïve individuals (48.3% vs. 16.7%).

Choi et al. [21] provided additional insight into the temporal relationship between PD-1 inhibitor exposure and ES by demonstrating that a shorter interval between checkpoint inhibitor therapy and ASCT was associated with both an increased risk and greater severity of ES. In their report, the most severe ES occurred in the patient with the shortest PD-1-to-transplant interval (20 days), whereas patients with longer intervals (64 and 91 days) experienced less severe manifestations. Importantly, absolute lymphocyte counts (ALC) measured at the time of stem cell collection and during engraftment did not correlate with ES severity: paradoxically, the patient with the lowest ALC developed the most severe clinical presentation. These observations suggest that lymphocyte quantity alone is not a reliable predictor of ES risk, whereas the timing of PD-1 inhibitor exposure relative to transplantation may play a more critical role. In our cohort, although the interval between the last PD-1 inhibitor dose and ASCT tended to be shorter in patients who developed severe, steroid-requiring pre-ES, this difference did not reach statistical significance. This likely reflects limited statistical power due to the small number of events rather than the absence of a true biological association, and is consistent with the hypothesis that residual immune activation from recent PD-1 blockade may predispose patients to exaggerated peri-engraftment inflammatory responses.

Consistent with prior reports of increased peri-engraftment inflammatory manifestations following PD-1 inhibitor exposure, Ibrahim et al. [22] described a very high incidence of ES in a small retrospective cohort of HL patients undergoing ASCT, with all patients fulfilling Maiolino-based criteria for ES at the time of neutrophil engraftment. Although the reported incidence was markedly higher than in larger series, the study predominantly captured ES manifestations occurring during the engraftment period and did not evaluate inflammatory events arising before neutrophil recovery, thereby reinforcing the concept that PD-1 inhibitor exposure substantially increases the burden of peri-engraftment inflammatory manifestations.

A precise evaluation of symptom timing in recently published case reports indicates that ES-like inflammatory manifestations may occur prior to neutrophil engraftment in PD-1-exposed patients. Demirsoy et al. [23] reported a nivolumab-treated HL patient who developed severe ES-compatible inflammatory manifestations on day +5 following ASCT, whereas neutrophil engraftment was documented on day +8. Although classified as ES, the temporal dissociation between symptom onset and neutrophil recovery supports reclassification of this event as pre-ES rather than classical post-engraftment ES.

Similarly, Sachin et al. [24] described a nivolumab-treated pediatric HL patient who developed a severe inflammatory syndrome on day +5 post-transplantation, clearly preceding neutrophil recovery and accompanied by marked cytokine elevation and multiorgan involvement. Despite being reported as ES, the early onset relative to engraftment, absence of neutrophil recovery at symptom onset, and rapid response to corticosteroid therapy are consistent with a pre-engraftment inflammatory phenotype. Collectively, these observations support the concept that PD-1 inhibitor exposure may shift ES-related inflammatory manifestations toward an earlier, pre-engraftment phase.

These observations are concordant with our findings, in which clinically significant, steroid-requiring pre-ES occurred exclusively before neutrophil engraftment and was restricted to patients with prior PD-1 inhibitor exposure. In both the published cases and our cohort, pre-ES was characterized by early onset, prominent systemic inflammation, exclusion of infectious etiologies, and a rapid response to corticosteroid therapy, supporting the notion that pre-ES represents a reproducible and clinically relevant inflammatory phenotype distinct from classical ES.

### 4.7. Integration of Current Findings with Biological Mechanisms

In our cohort, no cases of overt ES were identified when applying the more stringent Spitzer criteria. In contrast, use of the broader Maiolino criteria resulted in the identification of ES in three patients (4.7%), none of whom required systemic corticosteroid therapy. These findings indicate that classical ES remains an infrequent complication among HL patients undergoing ASCT. Notably, an additional three patients (4.7%) fulfilled both Spitzer and Maiolino diagnostic criteria but developed inflammatory symptoms prior to neutrophil engraftment, consistent with a diagnosis of pre-ES. All pre-ES cases necessitated systemic corticosteroid treatment following exclusion of infectious etiologies, underscoring the clinical significance of inflammatory manifestations occurring outside the conventional engraftment window. Importantly, while ES-compatible inflammatory features were relatively common when broader diagnostic definitions were applied, clinically significant disease requiring therapeutic intervention was restricted to a limited subset of patients. This observation highlights the importance of distinguishing transient, self-limiting inflammatory reactions from severe, clinically relevant manifestations that warrant prompt recognition and treatment.

In our cohort, all steroid-requiring pre-ES cases occurred in patients previously exposed to PD-1 inhibitors, and a statistically significant association between PD-1 inhibitor therapy and Spitzer-defined pre-ES was observed despite the limited sample size. The estimated relative risk and large absolute risk difference further support a clinically meaningful relationship. Although we explored the potential contribution of stem cell product characteristics, including MNC dose and the ratio of MNC to CD34+ cells, no significant associations were identified. These negative findings suggest that quantitative graft composition alone may be insufficient to capture the functional immune alterations induced by prior checkpoint inhibition, and should be interpreted cautiously given the small number of events. To contextualize our findings, published reports of ES and peri-engraftment inflammatory complications following ASCT in PD-1-exposed HL patients are compiled in Table 6.

A plausible biological explanation for the observed association between prior PD-1 inhibitor exposure and clinically significant pre-ES may involve the prolonged pharmacokinetics and sustained immunologic effects of PD-1 blockade. However, it is important to emphasize that this hypothesis remains speculative and is not directly supported by mechanistic data from our study. In the absence of detailed immunophenotyping or cytokine profiling, our interpretation is based on indirect clinical observations—such as the temporal association with PD-1 exposure and elevated CRP levels—and on previously published literature describing sustained immune activation following checkpoint inhibition.

Pembrolizumab and nivolumab have long elimination half-lives of approximately 26 and 40 days, respectively, and persistent drug exposure with ongoing immune activation has been documented for several months—and in some cases up to one year—after treatment discontinuation. This prolonged presence of PD-1 inhibitors may result in sustained T-cell activation and altered cytokine profiles that extend into the peri-engraftment period. Such a state of residual immune activation may critically influence the immunologic milieu during neutrophil recovery. Persistent T-cell activation, in combination with the influx of immune-competent MNC reinfused with the autologous stem cell graft, could amplify inflammatory signaling and lower the threshold for exaggerated immune responses. This mechanism may be particularly relevant in patients who receive multiple cycles of PD-1 blockade or proceed to transplantation after a short interval, resulting in higher residual PD-1 inhibitor exposure at the time of stem cell infusion [25,26].

Taken together, our results suggest that prior PD-1 inhibitor exposure may be associated with an increased risk of clinically significant pre-ES. These events may closely mimic infectious complications and therefore risk delayed recognition. From a practical standpoint, any early, unexplained inflammatory deterioration in PD-1-exposed patients during the first post-transplant days should prompt consideration of pre-ES and early initiation of corticosteroid therapy once infection has been reasonably excluded, as timely treatment may be life-saving.

To our knowledge, this study represents one of the first HL-focused analyses systematically evaluating inflammatory events occurring before neutrophil engraftment and linking steroid-requiring pre-ES to prior PD-1 exposure using both clinical and biomarker data.

### 4.8. Limitations

This study has several limitations inherent to its retrospective, single-center design. The relatively small sample size—particularly the low number of steroid-requiring events—limits statistical power and precludes multivariable analysis. In addition, inflammatory cytokine profiling and immunophenotypic analyses were not routinely available, restricting mechanistic insight into the observed associations. Only CRP was consistently measured as part of standard clinical care, which represents a nonspecific marker of inflammation and does not allow detailed characterization of the underlying immune processes. More informative biomarkers—such as IL-6, IL-10, TNF-α, and T-cell activation markers—were not available in this retrospective cohort. Notably, cytokine measurements, including IL-6, have recently been introduced into our institutional practice and are now being collected prospectively in patients undergoing ASCT, which may allow more precise characterization of the immunological mechanisms underlying pre-ES in future studies. Finally, variations in timing and duration of PD-1 inhibitor exposure, as well as supportive care practices, may have influenced outcomes and limit generalizability.

## 5. Conclusions

In conclusion, ES after ASCT remains uncommon in patients with HL; however, pre-ES is frequent when broader diagnostic criteria are applied. Clinically significant, steroid-requiring pre-ES was observed only among patients previously exposed to PD-1 inhibitors; however, given the very small number of clinically significant events (*n* = 3), statistical power was limited and the resulting effect estimates may be inflated. Accordingly, these findings should be interpreted as hypothesis-generating rather than as evidence of a causal relationship. Awareness of this potential association is clinically relevant, as early recognition and timely initiation of corticosteroid therapy—after careful exclusion of infectious causes—may prevent severe complications. Prospective studies are warranted to better define risk factors, optimal monitoring strategies, and preventive approaches in this high-risk population.

## Figures and Tables

**Table 1:** Diagnostic criteria for engraftment syndrome (adapted from Spitzer and Maiolino [6,7]).

SPITZER CRITERIA (2001) [6] (All 3 Major Criteria or 2 Major Criteria and 1 or More Minor Criteria Occurring Within 96 h of Engraftment)	MAIOLINO CRITERIA (2003) [7] (24 h Prior or Any Time After Engraftment)
**MAJOR CRITERIA** 1. Temperature > 38.3 °C with no identifiable infectious etiology. 2. Erythrodermatous rash involving > 25% of body surface area and not attributable to medications. 3. Noncardiogenic pulmonary edema, manifested by diffuse pulmonary infiltrates and hypoxia.	Noninfectious fever and either of these: rash, pulmonary infiltrates or diarrhea (at least 2/day)
**MINOR CRITERIA** 1. Hepatic dysfunction with either bilirubin ≥ 2 mg/dL or transaminase levels ≥ 2 times normal. 2. Renal insufficiency (serum creatinine) ≥ 2 times baseline. 3. Weight gain ≥ 2.5% of baseline body weight. 4. Transient encephalopathy unexplainable by other causes.	

**Table 2:** Baseline demographic, disease, and treatment characteristics of the study cohort (*n* = 64).

Characteristic	Value	Characteristics	Value	Characteristics	Value
**Patient characteristics**		**Treatment before ASCT**		**Disease status at ASCT**	
age at ASCT, years (median, range)	32 (18–62)	first-line therapy: ABVD, *n* (%)	63 (98.4)	complete metabolic remission (CMR), *n* (%)	54 (84.4)
male, *n* (%)	33 (51.6)	first-line therapy: BV + AVD, *n* (%)	1 (1.6)	partial remission (PR), *n* (%)	9 (14.1)
female, *n* (%)	31 (48.4)	salvage therapy: DHAP, *n* (%)	64 (100)	stable disease (SD), *n* (%)	1 (1.6)
**Disease characteristics**		BV-based salvage therapy, *n* (%)	34 (53.1)		
primary refractory disease, *n* (%)	24 (37.5)	PD-1 inhibitor exposure, *n* (%)	8 (12.5)		
relapsed disease, *n* (%)	40 (62.5)	time from last PD-1 dose to ASCT, days (median, range)	41 (30–146)		

ASCT: autologous stem cell transplantation; ABVD: doxorubicin, bleomycin, vinblastine, dacarbazine; AVD: doxorubicin, vinblastine, dacarbazine; BV: brentuximab vedotin; DHAP: dexamethasone, high-dose cytarabine, cisplatin; PD-1: programmed cell death protein 1; CMR: complete metabolic remission; PR: partial remission; SD: stable disease.

**Table 3:** Clinical and transplant characteristics of pre-engraftment syndrome (pre-ES) status.

	Severe Pre-ES	Non-Severe Pre-ES	Non-Pre-ES
**patients, *n* (%)**	3 (4.7)	31 (48.4)	30 (46.9)
**female, *n* (%)**	0 (0)	14 (21.9)	17 (26.6)
**male, *n* (%)**	3 (4.7)	17 (26.6)	13 (20.3)
**received PD-1 inhibitor therapy, *n* (%)**	3 (4.7)	1 (1.6)	2 (3.1)
**CMR before ASCT, *n* (%)**	1 (1.6)	26 (40.6)	27 (42.2)
**median infused CD34+ cell dose (×10^6^/kg)**	7.88 (3.63–7.91)	5.08 (2.54–8.44)	5.36 (2.54–8.56)
**median infused MNC dose (×10^8^/kg)**	1.67 (1.51–6.4)	3.3 (0.12–16.6)	n.a.
**symptom onset (days)**	4	5 (2–9)	n.a.
**neutrophil engraftment (days)**	10 (8–17)	9 (6–14)	9 (6–17)

pre-ES: pre-engraftment syndrome; PD-1: programmed cell death protein 1; ASCT: autologous stem cell transplantation; MNC: mononuclear cell; CMR: complete metabolic remission; n.a.: not available.

**Table 4:** Incidence and clinical characteristics of engraftment syndrome (ES) in heterogeneous hematopoietic stem cell transplantation (HSCT) cohorts [4,5,6,11,12,13,14].

Study	Period	Population	HL Patients (*n*)	ES Cases (*n*)	Median Engraftment (Days)	Median Symptom Onset (Days)	ES	Pre-ES	PD-1 Treatment (*N*)	PD-1-Exposed ES Cases (*n/N*)	Median CD34+ Cell Dose (×10^6^/kg)	Median MNC Dose (×10^8^/kg)	Corticosteroid Treatment	OS/PFS
**Cornell et al., 2015** [4]	n.a.	n.a. (review)	n.a.	n.a.	n.a.	n.a.	yes	no	n.a.	n.a.	n.a.	n.a.	yes	n.a.
**Maqbool et al., 2022** [11]	**n.a.**	**n.a. (review)**	**n.a.**	**autologous: 8–50%; allogeneic: 10–77%**	n.a.	n.a.	yes	no	n.a.	n.a.	n.a.	n.a.	yes	n.a.
**Spitzer, 2001** [6]	**n.a.**	**n.a. (criteria)**	**n.a.**	**n.a.**	±96 h	n.a.	yes	no	n.a.	n.a.	n.a.	n.a.	n.a.	n.a.
**Poonsombudlert et al., 2020** [5]	**–**	**meta-analysis (allogeneic transplantation)**	**n.a.**	**ES ↗ aGVHD (OR ~2.76)**	n.a.	n.a.	yes	no	n.a.	n.a.	n.a.	n.a.	n.a.	n.a.
**Lee et al., 2008** [12]	**1997–2004**	**50 total (allogeneic/cord blood transplantation—pediatric)**	**0**	**7 ES** **aplastic anemia (*n* = 3), AML (*n* = 2), CML (*n* = 1), Wiskott–Aldrich syndrome (*n* = 1)**	11	7 (5–8)	no	yes pre-ES after cord blood and bone marrow transplantation (n = 7); none after peripheral blood stem cell transplantation	n.a.	n.a.	1.66 (0.47–4.8)	4.41 (3.14–6.21)	yes	pre-ES 71.4% vs. non-pre-ES 49.6% (*p* = 0.417)
**Capizzi et al., 2001** [13]	1987–1998	146 total (autologous transplantation)	n.a.	19 PERDS lymphoma (*n* = 10), MM (*n* = 8), other (*n* = 1)	11 (8–25)	11 (4–25)	yes	no	n.a.	n.a.	n.a.	1.8 (0.74–4.4)	yes	n.a.
**Dispenzieri et al., 2008** [14]	1999–2007	30 total (autologous transplantation)	n.a.	5 POEMS	16 (15–18)	9 (8–11)	yes (27–47%, definition-dependent)	yes (symptom onset outside Maiolino/Spitzer time window)	n.a.	n.a.	4.46 (2.39–15.7)	6.86 (1.21–21.6)	yes	n.a.

HL: Hodgkin lymphoma; ES: engraftment syndrome; pre-ES: pre-engraftment syndrome; PD-1: programmed cell death protein 1; MNC: mononuclear cell; OS: overall survival; PFS: progression-free survival; aGVHD: acute graft-versus-host disease; PERDS: peri-engraftment respiratory distress syndrome; AML: acute myeloid leukemia; CML: chronic myeloid leukemia; MM: multiple myeloma; POEMS: polyneuropathy, organomegaly, endocrinopathy, monoclonal gammopathy, and skin changes syndrome; OR: odds ratio; n.a.: not available. The arrow (↗) indicates a positive association (increased risk).

**Table 5:** Incidence and clinical characteristics of engraftment syndrome (ES) in mixed-diagnosis hematopoietic stem cell transplantation (HSCT) cohorts including Hodgkin lymphoma (HL) subpopulations [7,15,16,17].

Study	Period	Population	HL Patients (*n*)	ES Cases (*n*)	Median Engraftment (days)	Median Symptom Onset (days)	ES	Pre-ES	PD-1 Treatment (*N*)	PD-1-Exposed ES Cases (*n/N*)	Median CD34+ Cell Dose (×10^6^/kg)	Median MNC Dose (×10^8^/kg)	Corticosteroid Treatment	OS/PFS
**Maiolino et al., 2003** [7]	1994–2000	125 total (autologous transplantation) MM (*n* = 45), HL (*n* = 37), NHL (*n* = 29), breast cancer (*n* = 6), AML (*n* = 5), germ cell tumor (*n* = 1), plasma cell leukemia (*n* = 1), amyloidosis (*n* = 1)	37	25 ES MM (*n* = 13), HL (*n* = 2), NHL (*n* = 6), breast cancer (*n* = 3)	11	n.a.	yes	no	n.a.	n.a.	ES 6.7 (1.1–22.7); non-ES 5.35 (1.1–30.7)	ES 3.68 (1.95–11.6) non-ES 4.2 (0.43–13.85)	yes	n.a.
**Bai et al., 2021** [15]	2015–2019	260 total; HL (*n* = 37); NHL (*n* = 223)	37	16 PERDS HL (*n* = 4), NHL (*n* = 12)	9 (7–11)	8 (6–10)	yes	no	pembrolizumab (*n* = 11), sintilimab (*n* = 14), toripalimab (*n* = 6)	8/31 (HL + NHL)	4.2 ± 2.7	n.a.	yes	n.a.
**Sheth et al., 2018** [16]	2006–2014	171 total; MM (*n* = 87), HL (*n* = 48), NHL (*n* = 34), other (*n* = 2)	48	46 ES MM (*n* = 23), HL (*n* = 16), NHL (*n* = 7)	11	n.a.	yes	no	n.a.	n.a.	3.3	n.a.	yes	n.a.
**Carreras et al., 2010** [17]	2001–2007	328 total (autologous transplantation) acute leukemia (*n* = 34), amyloidosis (*n* = 25), POEMS (*n* = 5), scleromyxedema (*n* = 1), MM (*n* = 89), NHL (*n* = 102), HL (*n* = 48), CLL/PL (*n* = 12), WM (*n* = 3), solid tumors (*n* = 4), AID (*n* = 2), CML (*n* = 2)	42	acute leukemia (*n* = 2), amyloidosis (*n* = 12), POEMS (*n* = 1), scleromyxedema (*n* = 1), MM (*n* = 10), NHL (*n* = 13), HL (*n* = 2), CLL/PL (*n* = 1)	11.5 (10–35)	n.a.	yes	no	n.a.	n.a.	3.6 ± 2.2	n.a.	yes	n.a.

HL: Hodgkin lymphoma; NHL: non-Hodgkin lymphoma: MM, multiple myeloma; AML: acute myeloid leukemia; CLL: chronic lymphocytic leukemia; PL: plasma cell leukemia; WM: Waldenström macroglobulinemia; CML: chronic myeloid leukemia; AID: autoimmune disease; ES: engraftment syndrome; pre-ES: pre-engraftment syndrome; PERDS: peri-engraftment respiratory distress syndrome; PD-1: programmed cell death protein 1; MNC: mononuclear cell; OS: overall survival; PFS: progression-free survival; n.a.: not available.

**Table 6:** Engraftment-related inflammatory complications after autologous stem cell transplantation (ASCT) following prior PD-1 inhibitor exposure in relapsed or refractory Hodgkin lymphoma (R/R HL) patients [18,19,20,21,22,23,24].

Study	Period	HL Patients (*n*)	ES Cases (*n*)	Median Engraftment (Days)	Median Symptom Onset (Days)	ES	Pre-ES	PD-1 Treatment (*N*)	PD-1-Exposed ES Cases (*n/N*)	PD-1 Treatment → ASCT (Days)	Median CD34+ Cell Dose (×10^6^/kg)	Median MNC Dose (×10^8^/kg)	Corticosteroid Treatment	OS/PFS
**Ibrahim et al., 2024** [22]	2017–2024	11	11	14 (10–20)	~14	yes	no	pembrolizumab (7), nivolumab (4)	11/11	n.a.	4.2 (3–5.1)	n.a.	yes	2-year PFS 73%, 2-year OS 82%
**Choi et al., 2026** [21]	2025	3	3	10 (10–11)	10 (9–11)	yes	no	nivolumab (2), pembrolizumab (1)	3/3	57 (20–91)	6.3 (2.74–6.3)	n.a.	yes (2/3)	n.a.
**Taranto et al., 2023** [18]	2015–2023	53	11	10	~10	yes	no	nivolumab (37), pembrolizumab (16)	11/53	n.a.	7.28	n.a.	yes (7/11)	2-year PFS 78%, 2-year OS 90%
**Odetola et al., 2023** [20]	2018–2023	47	17	10 (8–12)	9 (2–11)	yes	no	commonly pembrolizumab (29)	14/29	n.a.	n.a.	n.a.	yes	OS 95.7%
**Sachin M P et al., 2025** [24]	2025	1 pediatric patient	1	**14**	**5**	no	**yes**	nivolumab (1)	1/1	n.a.	n.a.	n.a.	yes	n.a.
**Demirsoy et al., 2025** [23]	2025	1	1	**8**	**5**	no	**yes**	nivolumab (1)	1/1	15	n.a.	n.a.	yes	n.a.
**Khouderchah et al., 2026** [19]	2014–2024	141	4	n.a.	n.a.	yes	n.a.	agent n.s. (50)	3/50	n.a.	6.18 (5.28–8.29)	n.a.	yes	n.a.
**Author’s cohort**	2018–2025	64	3	**10 (8–17)**	**4**	no	**yes**	pembrolizumab (6), nivolumab (2)	3/8	37 (30–87)	7.88 (3.63–7.91)	1.67 (1.51–6.4)	yes	5-year PFS 87%, 5-year OS 90%

HL: Hodgkin lymphoma; ES: engraftment syndrome; pre-ES: pre-engraftment syndrome; PD-1: programmed cell death protein 1; ASCT, autologous stem cell transplantation; MNC: mononuclear cell; OS: overall survival; PFS: progression-free survival; n.a.: not available; n.s.: not specified.

## Data Availability

Data sets generated during the current study are available from the corresponding author on reasonable request.

## Abbreviations

The following abbreviations are used in this manuscript:

**Abbreviation**	**Full term**
ABVD	doxorubicin, bleomycin, vinblastine, dacarbazine
AID	autoimmune disease
ALC	absolute lymphocyte count
AML	acute myeloid leukemia
ASCT	autologous stem cell transplantation
aGVHD	acute graft-versus-host disease
AVD	doxorubicin, vinblastine, dacarbazine
BEAM	carmustine, etoposide, cytarabine, melphalan
BV	brentuximab vedotin
CLL	chronic lymphocytic leukemia
CML	chronic myeloid leukemia
CMR	complete metabolic remission
CMV	cytomegalovirus
CRP	C-reactive protein
CRS	cytokine release syndrome
CT	computed tomography
DHAP	dexamethasone, high-dose cytarabine, cisplatin
ES	engraftment syndrome
EBV	Epstein–Barr virus
G-CSF	granulocyte colony-stimulating factor
GVD	gemcitabine, vinorelbine, pegylated liposomal doxorubicin
GVHD	graft-versus-host disease
HL	Hodgkin lymphoma
HSCT	hematopoietic stem cell transplantation
ICE	ifosfamide, carboplatin, etoposide
ICI	immune checkpoint inhibitor
IFN-γ	interferon-gamma
IL	interleukin
irAE	immune-related adverse event
MNC	mononuclear cell
MM	multiple myeloma
NHL	non-Hodgkin lymphoma
OS	overall survival
PD-1	programmed cell death protein 1
PERDS	peri-engraftment respiratory distress syndrome
PCR	polymerase chain reaction
PET/CT	positron emission tomography/computed tomography
PFS	progression-free survival
PL	plasma cell leukemia
POEMS	polyneuropathy, organomegaly, endocrinopathy, monoclonal gammopathy, and skin changes syndrome
pre-ES	pre-engraftment syndrome
R/R	relapsed or refractory
TNF-α	tumor necrosis factor-alpha
WM	Waldenström macroglobulinemia
